# Analysis of the pathogenicity and pathological characteristics of *NOTCH3* gene-sparing cysteine mutations *in vitro* and *in vivo* models

**DOI:** 10.3389/fnmol.2024.1391040

**Published:** 2024-12-20

**Authors:** Zhenping Gong, Wan Wang, Ying Zhao, Yadan Wang, Ruihua Sun, Haohan Zhang, Fengyu Wang, Yaru Lu, Jiewen Zhang

**Affiliations:** ^1^Department of Neurology, Henan Province People’s Hospital, Xinxiang Medical University, Zhengzhou, China; ^2^Department of Neurology, Zhengzhou University People’s Hospital, Henan Province People’s Hospital, Zhengzhou, China; ^3^Department of Neurology Henan University People’s Hospital, Henan Province People’s Hospital, Zhengzhou, China; ^4^Academy of Medical Sciences, Zhengzhou University, Zhengzhou, China

**Keywords:** CADASIL, cysteine-sparing NOTCH3 mutation, NOTCH3 ECD, *in vitro* cell model, *in vivo* knock-in mice model

## Abstract

**Background:**

Cerebral autosomal dominant arteriopathy with subcortical infarcts and leukoencephalopathy (CADASIL) is one of the most common inherited cerebral small vessel diseases caused by the NOTCH3 gene mutation. This mutation leads to the accumulation of NOTCH3 extracellular domain protein (NOTCH3^ECD^) into the cerebral arterioles, causing recurrent stroke, white matter lesions, and cognitive impairment. With the development of gene sequencing technology, cysteine-sparing mutations can also cause CADASIL disease, however, the pathogenicity and pathogenic mechanisms of cysteine-sparing mutations remain controversial.

**Objective:**

To analyze the pathogenicity and pathological features of cysteine-sparing mutations in both *in vitro* and *in vivo* mouse models.

**Methods:**

A cysteine-sparing mutant of NOTCH3^ECD^ R75Q was constructed by lentiviral transfection *in vitro*, and the *NOTCH3 R75Q* knock-in mouse model was constructed by CRISPR/Cas-mediated genome engineering *in vivo*. A cycloheximide pulse-chase experiment was used to analyze the degradation of NOTCH3 extracellular domain proteins, and the deposition characteristics of NOTCH3^ECD^ were quantitatively analyzed by immunohistochemical staining. The characteristics of the smooth muscle cells and granular osmiophilic materials were observed using electron microscopy.

**Results:**

We elucidated that the *NOTCH3 R75Q* mutation is pathogenic. NOTCH3^ECD^ R75Q was found to be resistant to protein degradation and more likely to cause abnormal aggregation of NOTCH3^ECD^, resulting in reduced cell activity *in vitro*. The *NOTCH3 R75Q* mouse model showed pathological characteristics of CADASIL, with age-dependent NOTCH3^ECD^, granular osmiophilic material, and degenerated smooth muscle cells detected in the brain.

**Conclusion:**

To our knowledge, this is the first study to analyze the pathogenicity of *NOTCH3 R75Q* cysteine-sparing mutations in both *in vitro* and *in vivo* models. We demonstrate that NOTCH3^ECD^ induced by *NOTCH3 R75Q* mutation has toxic effects on cells and reveal the deposition characteristics of NOTCH3^ECD^ in the brain. This provides a feasible model and lays the foundation for further studies on the pathogenesis and therapeutic strategies of *NOTCH3* cysteine-sparing mutations.

## 1 Introduction

Cerebral autosomal-dominant arteriopathy with subcortical infarction and leukoencephalopathy (CADASIL) is the most commonly reported hereditary cerebral small vessel disease globally and is closely associated with ischemic stroke, leukoencephalopathy, and vascular cognitive impairment (Joutel et al., 1996; Chabriat et al., 2009; Rutten et al., 2016). CADASIL progresses slowly and gradually, with initial clinical symptoms appearing as early as 30–40 years of age, but usually onset in middle age, characterized by recurrent stroke, migraine, cognitive decline, and emotional disorders mainly characterized by apathy and depression (Opherk, 2004; Dichgans et al., 1998; Markus et al., 2002; Viswanathan et al., 2007; Reyes et al., 2009; Charlton et al., 2006). Magnetic resonance imaging (MRI) typically shows recent small subcortical infarcts, lacunar infarctions, and white matter hyperintensities (WMH), especially in the anterior temporal pole and external capsule, with or without microbleeds and enlarged perivascular spaces (Wang, 2018; Pantoni, 2010; Di Donato et al., 2017; Jouvent et al., 2020). In addition, the gold standard for diagnosis includes *NOTCH3* gene mutations found by genetic testing of patients, and positive staining for granular osmiophilic material (GOM) or NOTCH3 protein detected by pathological testing (Chabriat et al., 1998).

The classical hypothesis is that the pathogenic mechanism of CADASIL involves an odd number of cysteines in 34 epidermal growth factor-like repeat (EGFR) regions of the NOTCH3 receptor. Unpaired cysteine residues can lead to EGFR misfolding and increased multimerization of NOTCH3 extracellular domain (NOTCH3^ECD^) proteins by disrupting normal disulfide bond formation, eventually leading to CADASIL (Joutel et al., 1996; Oliveira et al., 2023; Chabriat et al., 2009). However, with the development of gene sequencing technology, cysteine-sparing mutations that do not affect the change in cysteine quantity have gradually emerged, although their pathogenicity is still controversial and their pathogenic mechanism remains unclear (Muiño et al., 2017). According to the Human Gene Mutation Database database, approximately 5% of mutations in *NOTCH3* related to CADASIL are cysteine-preserving missense mutations (Ferrante et al., 2019). Some researchers found that *NOTCH3* D80G mutation carriers in four families showed typical neuroimaging and clinical phenotypes of CADASIL (Wollenweber et al., 2015). Other researchers have found that CADASIL and R75P mutations are more common in Japan and South Korea, and patients with this mutation show WMHs in the anterior temporal pole and NOTCH3^ECD^ deposition on the vascular wall (Ueda et al., 2015). In addition, a patient with the R75Q mutation has been reported for the first time in China, exhibiting clinical manifestations including stroke, cognitive impairment, affective disorder, and gait disorder. Neuroimaging of the patient showed a lacunar infarction and extensive WMHs involving the temporal pole and external capsule, and a skin biopsy showed a positive GOM (Zhang et al., 2020), which is an abnormal protein aggregate containing the extracellular domain of NOTCH3 (NOTCH3^ECD^), and located around smooth muscle cells and the basement membranes of arteriolar vessels (Baudrimont et al., 1993). Moreover, several cysteine-sparing mutations (R61W, R75P, D80G, and R213K) may be potentially pathogenic because they have typical clinical CADASIL characteristics and extensive WMHs without other potential pathogenic mutations, as well as positive GOM on skin biopsy (Muiño et al., 2017).

However, the current research is limited to a summary of clinical cases, and there are relatively few *in vitro* experimental studies. In some studies, *in vitro* cellular models of noncysteine mutations have been constructed. Wollenweber et al. used a single-particle assay to demonstrate that the aggregation of D80G mutants *in vitro* was consistent with that of cysteine mutations (Wollenweber et al., 2015). Huang et al. used plasmids to transfect G73A and R75P mutants expressing the NOTCH3 protein (Huang et al., 2020), and Liu et al. used a lentivirus to transfect the NOTCH3^ECD^ A564T mutant (Liu et al., 2022). Both studies found abnormal aggregation of NOTCH3 and increased protein degradation resistance in cysteine-sparing mutants, further illustrating the potential pathogenic role of the cysteine-sparing mutation; therefore, NOTCH3 can also be used as a pathogenic marker of great significance in the study of cysteine-sparing mutations. In contrast, there are currently only CADASIL mouse models with cysteine mutations and not cysteine-sparing mutations. This limits the analysis of the pathogenicity of cysteine-sparing mutations as well as the study of pathogenic mechanisms.

In summary, there have been relatively few *in vivo* and *in vitro* studies on cysteine-sparing mutations. Therefore, this study constructed a *NOTCH3 R75Q* cysteine-sparing mutation in an *in vitro* cell model and *in vivo* transgenic mouse model. NOTCH3 was used as a biomarker to evaluate the pathogenicity of the mutation and preliminarily explore the relevant mechanism of the cysteine-sparing mutation.

## 2 Methods

### 2.1 Cell culture and lentivirus transfection

Human embryonic kidney (HEK) 293 T cells (Shanghai, China) were cultured in Dulbecco’s modified Eagle’s medium supplemented with 10% fetal bovine serum at 37°Cin 5% CO2. Next, 293 T cells were transfected with lentivirus (BrainVTA, China) expressing WT (rLV-CMV- Flag-NOTCH3-P2A-Puro- WPRE), R75Q (rLV-CMV-Flag-NOTCH3(R75Q)-P2A-Puro-WPRE), and the empty vector (rLV-CMV -Flag-P2A-Puro-WPRE) to establish stable transfected cell lines. A total of 6 × 10^4^ 293 T cells/well were prepared in a 24-wells plate. The following day, the cells in each well were transduced with lentivirus at a multiplicity of infection (MOI) of 1. After 24 h, the transduction media was removed and replaced with fresh DMEM added 10% FBS. Subsequently, the transduced cells were selected using the antibiotic puromycin (2 μg/mL) (Solarbio, China) and passaged once every three days in a ratio of 1:4.

### 2.2 Cellular immunofluorescence

Appropriate densities (5–10 × 10^4^) of WT and R75Q cells were seeded in 24-well plates, fixed with 4% PFA for 30 min, broken with 0.3% Triton for 20 min, and blocked with 5% bovine serum albumin (BSA) for 1 h. After addition of Anti-NOTCH3/N3ECD antibody (Sigma, United States) diluent (1:1000), the cells were incubated at 4°C, and then stained with Goat Anti-Mouse IgG H&L (Alexa Fluor^Ⓡ^594) antibody (abcam, UK) diluent (1:1000). Finally, anti-fluorescence quenching agent containing DAPI (Sigma, United States) was added to seal the slides and observed under a fluorescence microscope (Olympus BX53FL Research fluorescence microscope), so that NOTCH3^ECD^ could show red fluorescence.

### 2.3 Cellular protein extraction and protein quantification

The cells were collected and added to RIPA protein lysate (containing 1% phosphatase-protease inhibitor), lysed by ultrasonic cell fragmentation apparatus, and centrifuged at low temperature (4°C, 12000 x g for 30 min). The supernatant from the centrifuge tube was used as the desired protein. Protein quantification was performed using the bicinchoninic acid (BCA) method (Thermo Scientific, United States), and absorbance at 562 nm was measured using a microplate spectrophotometer (Synergy H1, BioTek Instruments, United States). The different proteins were diluted to the same concentration with double distilled water and 5 × sodium dodecyl sulfate loading buffer, boiled in water for 5 min, and stored at −80°C.

### 2.4 Western blotting

According to the molecular weight of the target protein, the concentration glue and separation glue were configured in the corresponding proportion, and the loading amount of each protein was 20 μg for electrophoresis. After electrophoresis, the membrane was transferred according to the molecular weight of the protein. After blocking with 5% skim milk or BSA solution on a shaker for 1 h at room temperature, the corresponding bands were cut according to the molecular weight of the target protein and internal reference. The bands were incubated with 1:1000 dilution of Anti-NOTCH3/N3ECD antibody (Sigma, United States) overnight at 4°C and 1:1000 dilution of Goat Anti-Mouse IgG H&L (HRP) (Beyotime, China) for 1 h at about 22°C.

### 2.5 Cell counting kit-8 assay

The density of cell spreading was approximately 5–10 × 10^4^, and after 24 h of culture, 10 μL of CCK8 (Cell counting kit-8) solution (Dojindo, Japan) was added and incubated for 4 h. The absorbance was measured at 450 nm using a microplate spectrophotometer (Synergy H1, BioTek Instruments, United States). Data were obtained from three independent *in vitro* experiments.

### 2.6 NOTCH3^ECD^ degradation assay

The cell density was approximately 5–10 × 10^5^. After 24 h of culture, the cells were treated with cycloheximide (concentration 150 μg/mL) for 0, 3, 6, and 9 h. Next, the cells were collected, and the total protein was extracted. The BCA (Thermo Scientific, United States) method was used for protein quantification, western blotting was used to detect the relative degradation level and stability of the target protein. Data were obtained from three independent *in vitro* experiments.

### 2.7 Construction of the *NOTCH3 R75Q* mouse model

Through CRISPR/Cas-mediated genome engineering, the “SM22α promoter-mutant human NOTCH3 CDS-P2A-EGFP-SV40 late pA” cassette was inserted into H11 (~0.7 kb 5′ of Eif4enif1 gene and ~ 4.5 kb 3′ of eif4enif1 gene). The BAC clone was used as a template to generate a homology arm by polymerase chain reaction (PCR) to construct a targeting vector. Cas9 and gRNA were coinjected with the targeting vector into fertilized mouse eggs for primary mouse production. These mice were genotyped by PCR, followed by sequencing analysis to identify mice with the *NOTCH3* R75Q mutation to examine germline transmission and the generation of F0 animals.

Gene knock-in mice appraisal designed primers:

PCR Primers WT (Annealing Temperature 60.0°C): Wild type allele: 479 bp.

F: 5’-CTCTACTGGAGGAGGACAAACTG-3′;

R: 5’-GTCTTCCACCTTTCTTCAGTTAGC-3′;

PCR Primers *R75Q* (Annealing Temperature 60.0°C): R75Q allele: 375 bp.

F: 5’-TGCTAACCATGTTCATGCCTTCTT-3′; R: 5’-CACTCATCCACGTCGCTTCG-3′;

### 2.8 Genetic identification of *NOTCH3 R75Q* mice

Mouse DNA was obtained from mouse tail tissues by lysis with Mouse Tissue Lysis Buffer (Vazyme, Nanjing, China) and proteinase K (Vazyme, Nanjing, China) for 30 min at 55°C. Primers targeting *NOTCH3* and DNA samples were used to prepare the PCR amplification system, and DNA was amplified by PCR. The amplified products were subjected to agarose gel electrophoresis, and the Tg mice were identified according to the bands at different positions (the mutant allele was highlighted at 375 bp, and the wild-type allele was highlighted at 479 bp).

### 2.9 Immunohistochemistry and immunofluorescence

Wild-type mice bred in the same litter as the Tg mice were selected as the control group, and homozygous mutant mice (Tg) were selected as the three experimental groups at 6, 10, and 20 months of age (*n* = 4). The brain and skin tissues of the mice were fixed by perfusion with a 0.9% NaCl solution and 4% paraformaldehye (PFA). After sampling, the tissues were refixed with glutaraldehyde and dehydrated with different concentrations of sucrose solution. The brain tissue was sectioned coronally at a thickness of 25 μm, ranging from bregma +2.00 mm to −5.00 mm, from after the fusion of the anterior commissural to the anterior vermis. Transverse sections of skin tissue were made at a thickness of 20 μm. Immunohistochemical and immunofluorescence staining for NOTCH3^ECD^ and smooth muscle cell (SMC) were performed. Membranes were cleaved with 3% Triton and blocked with 10% normal goat serum (NGS) for 1 h. Anti-NOTCH3/N3ECD (Sigma, United States), Anti-NOTCH3 antibody (abcam, UK), transgelin/SM22 Polyclonal antibody (proteintech, United States) and Rb X Olig-2 Polyclonal Antibody (Merckmillipore, United States) were diluted 1:500 in 1 × PBS, and brain and skin tissues were incubated overnight at 4°C. Goat Anti-Mouse IgG H&L (Alexa Fluor^Ⓡ^594) antibody (abcam, UK), were diluted 1:200 in 1 × PBS and incubated for 1.5 h at about 22°C (in the dark). For fluorescence staining, the nuclei were stained with Hoechst 33258 (Biofount, China) and the slides were sealed with an anti-fluorescence quenching agent. Red fluorescence was observed under a fluorescence microscope. Immunohistochemical staining was performed using diaminobenzidine (DAB) (Beyotime, China) or alkaline phosphatase (ALP) (Beyotime, China). The tan (DAB) and blue-purple (ALP) areas were observed under a light microscope (Olympus BX53-P polarizing microscope). DyLight 594 labeled *Lycopersicon Esculentum* (Tomato) Lectin (LEL, TL) (DL-1177-1) (Thermo, United States) and One Step TUNEL Apoptosis Assay Kit (Beyotime, China) were used to lectin and TUNEL staining.

### 2.10 Electron microscopy analysis

Tissues were prepared for immunohistochemistry (IHC) and immunofluorescence (IF). Brain tissues were fixed with 4% PFA and glutaraldehyde, fixed in 1% buffered osmium tetroxide, dehydrated in increasing grades of ethanol, treated with propylene oxide, and embedded in an epoxy resin. Sections (80 nm) were prepared using an ultramicroscope (Leica EMUC7), double-stained with uranyl acetate and lead citrate, and viewed under a transmission electron microscope (TEM HITACHI HT7700 120kv).

### 2.11 Analysis and statistics

WB was performed using ImageLab (version 6.1) and ImageJ software to analyze the gray value of the target protein and internal reference, and the relative expression was calculated. IHC, IF, and transmission electron images were captured using 3DHISTECH’s Slide Converter, CaseViewer, and Image Pro Plus (version 6.0) software. Images of each group and strategy maps were drawn using Adobe Illustrator 2023 software.

We used Image Pro Plus software quantitative NOTCH3^ECD^ and SMC. For *in vitro* cellular immunofluorescence, the ratio between the Integrated Optical Density (IOD) of NOTCH3^ECD^ expressing red fluorescence and the IOD of DAPI expressing blue nucleus was used as the deposition rate of NOTCH3^ECD^. The deposition rate of NOTCH3 was used to represent the amount of NOTCH3 expression in cells *in vitro*. The deposition rate of NOTCH3^ECD^ was calculated by the ratio of the brown NOTCH3^ECD^ positive area (Area) to the blue SMC coverage area (Area). The degree of NOTCH3^ECD^ was defined as the ratio of the integrated density of NOTCH3^ECD^ to the Area involved in NOTCH3^ECD^. The positive staining area of SMC was used as the standard to quantify the number of vascular smooth muscle cells (VSMCs).

SPSS (version 26.0) and GraphPad Prism (version 8.0) software were used for the statistical analysis of the data, and statistical plots were drawn using GraphPad Prism (version 8.0). The *SEM* value of the mean was used to represent continuous variables, and the differences between the two groups were compared using the two-tailed Student’s t test or Mann–Whitney U test. Differences among three or more groups were compared using Analysis of Variance (ANOVA) or Kruskal–Wallis *H* test, and Least-Significant Difference (*LSD*) was used for pairwise comparisons. Pearson’s correlation coefficient was used to analyze the correlations between variables. *p <* 0.05 was considered statistically significant.

## 3 Results

### 3.1 Abnormal aggregation of NOTCH3^ECD^ R75Q *in vitro*

**Figure 1:**
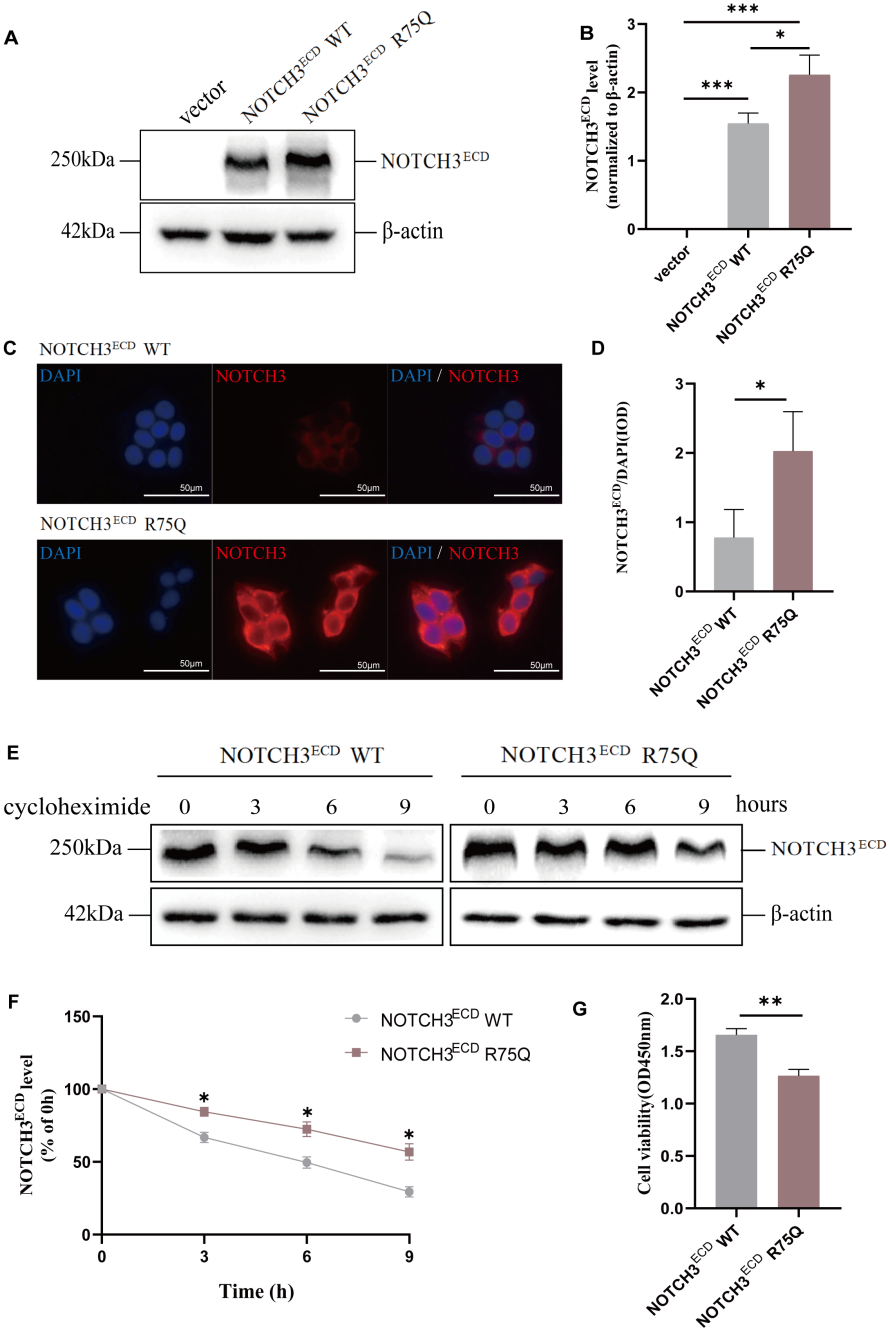
Abnormal expression and degradation of NOTCH3^ECD^ in cells: **(A)** NOTCH3^ECD^ protein levels are detected by western blot, and *β*-actin is used as a loading control. **(B)** Quantitative analysis of **A**. **(C)** Immunofluorescence staining shows NOTCH3^ECD^ (red) and nuclei (blue). **(D)** Semi-quantitative analysis of **C**. Analysis is performed using image pro plus software to quantify the integrated optical density (IOD) parameters. The ratio of IOD (NOTCH3^ECD^) to IOD (nucleus) is used to quantify the deposition rate of NOTCH3^ECD^. **(E)** Analysis of protein degradation in NOTCH3^ECD^ WT group and NOTCH3^ECD^ R75Q group. Stably transduced HEK 293 T cells are chased with 150 g/mL cycloheximide (CHX) at different time points and detected by immunoblotting, and β-actin is used as a loading control. **(F)** Quantitative analysis of **E**. Data are presented as means ± SD. ∗*p* < 0.05, *n* = 3 per group. **(G)** Quantification of cell viability in the NOTCH3^ECD^ WT and NOTCH3^ECD^ R75Q groups. One—way ANOVA or Kruskal–Wallis *H* test, and Least-Significant Difference (*LSD*) was used for pairwise comparisons.

To further investigate the pathogenicity of the cysteine-sparing mutation site, we constructed an *in vitro* human *NOTCH3 R75Q* mutation-site viral vector that was used to transfect 293 T cells to construct a stable lentiviral cell line. 293 T cells were transfected with lentivirus (BrainVTA, Wuhan, China) expressing WT (rLV-CMV- Flag-NOTCH3-P2A-Puro- WPRE), R75Q (rLV-CMV-Flag- NOTCH3(R75Q)-P2A-Puro-WPRE), and the empty vector (rLV-CMV -Flag-P2A-Puro-WPRE) to establish stable transfected cell lines. Western blotting showed that NOTCH3^ECD^ protein expression in the NOTCH3^ECD^ R75Q group was significantly higher than in the empty vector and NOTCH3^ECD^ WT group (*p* < 0.05) (Figures 1A,B). Cellular immunofluorescence staining showed that the NOTCH3^ECD^ was located in the cytoplasm around the nucleus, and NOTCH3^ECD^ R75Q had a larger positive area in the cytoplasm than NOTCH3^ECD^ WT, showing a more obvious perinuclear aggregation (*p* < 0.05) (Figures 1C,D). To further assess the toxic effects of NOTCH3^ECD^ in cells, CCK-8 cell viability assay was performed to examine cell viability in different groups. The results showed that the cell viability of NOTCH3^ECD^ R75Q group was lower than that of NOTCH3^ECD^ WT group (*p* < 0.05) (Figure 1G). The NOTCH3^ECD^ degradation assay further showed that NOTCH3^ECD^ in the mutant group was more resistant to protein degradation (*p* < 0.05) (Figures 1E,F). These results indicate that NOTCH3^ECD^ R75Q is resistant to protein degradation; therefore, it is more likely to cause abnormal accumulation of NOTCH3^ECD^, resulting in decreased cell activity.

### 3.2 Construction of a gene knock-in mouse model with the *NOTCH3 R75Q* mutation

**Figure 2:**
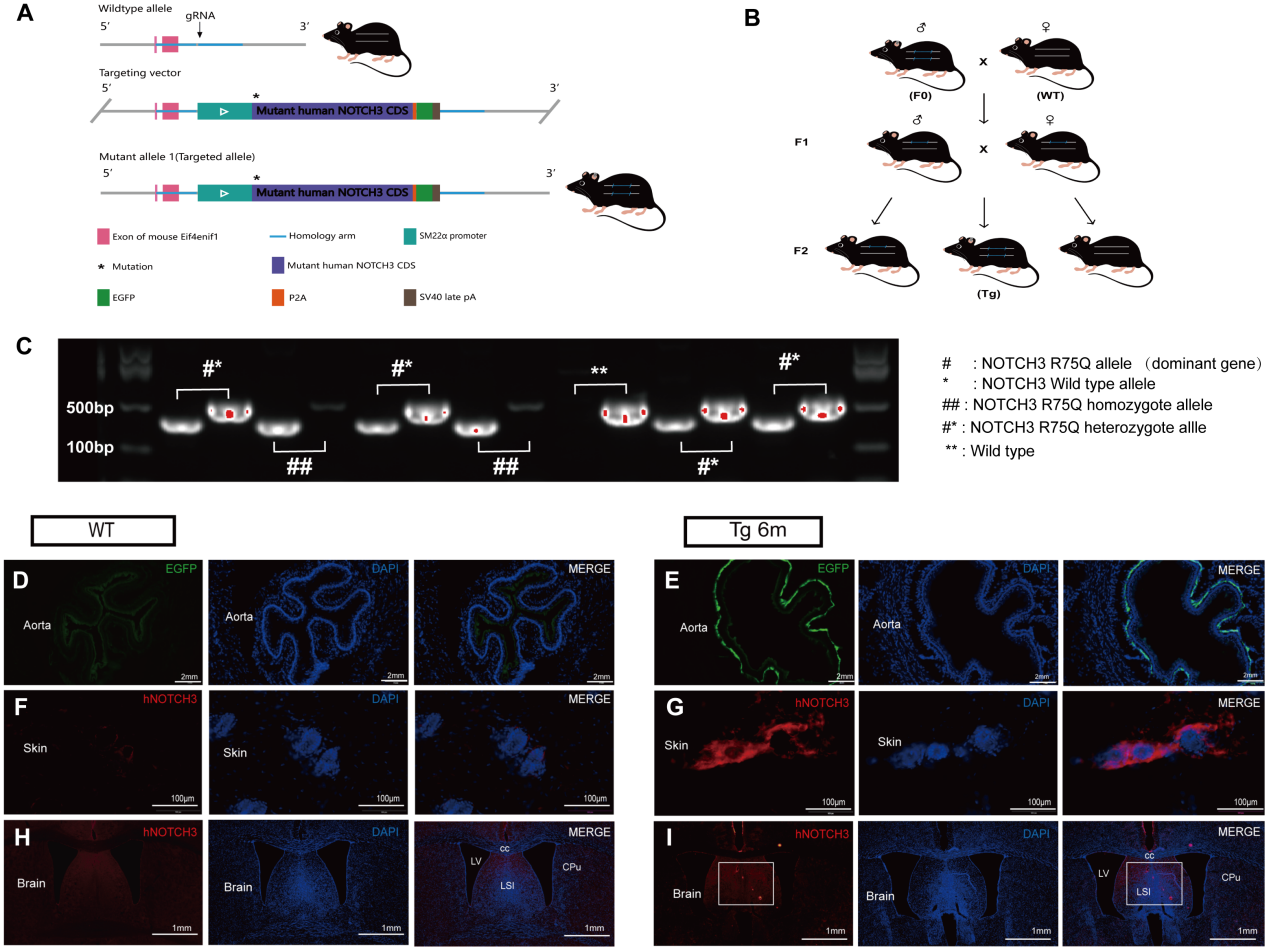
Construction and validation of gene knock-in mouse model: **(A)** Gene knock-in strategies in model mice. **(B)** Cultivation and reproduction of transgenic mice. **(C)** Genotype identification is performed by polymerase chain reaction (PCR). ## stands for homozygote, #* stands for heterozygote, and ** stands for wild type. **(D,E)** Immunofluorescence staining exhibits smooth muscle cell (SMC) carrying the EGFP label (green) and nuclei (blue). **(F,G)** Immunofluorescence staining exhibits NOTCH3^ECD^ (red), and nuclei (blue) in Transgenic mouse skin. **(H,I)** Immunofluorescence staining exhibits NOTCH3^ECD^ (red), and nuclei (blue) in transgenic mouse brain.

To further investigate the *in vivo* pathogenicity and mechanism of the cysteine-sparing *NOTCH3 R75Q* mutation, we generated a *NOTCH3 R75Q* transgenic mouse model by using *CRISPR/cas9* gene knock-in technology to knock in the human *NOTCH3 R75Q* mutation at *H11* site in C57BL/6 J mice. Then, homozygous transgenic mice of the F0 generation with the *NOTCH3 R75Q* gene mutation were obtained (Figure 2A). F0 and WT mice were bred as heterozygous transgenic mice of the F1 generation, and the heterozygotes of the F1 generation were interbred and then bred as Tg mice (Figure 2B). To obtain homozygous Tg mice, the genotypes of the mice were identified using PCR. The highlighted band present only at 325 bp represents homozygous Tg mice, the highlighted band present only at 479 bp represents WT mice, and one highlighted band present at each of the two positions represents heterozygous mice. According to the identification results, homozygous Tg mice were selected as the experimental group (Figure 2C). For further verification, we observed EGFP green fluorescence (Figures 2D–E) expression in the aorta of Tg mice under fluorescence microscopy, which proved that the knocked-in human *NOTCH3* gene could be expressed in the vascular smooth muscle cells of the mice. As shown by immunofluorescence staining, *NOTCH3* extracellular domain protein was stained red and aggregated in the skin and brain of Tg mice, which further confirmed that the mutant *NOTCH3* gene was expressed in both the peripheral and central tissues of Tg mice (Figures 2F–I).

### 3.3 *NOTCH3 R75Q* mice presenting pathological features of CADASIL

#### 3.3.1 Characteristics of NOTCH3 ECD deposition in R75Q mouse brain

**Figure 3:**
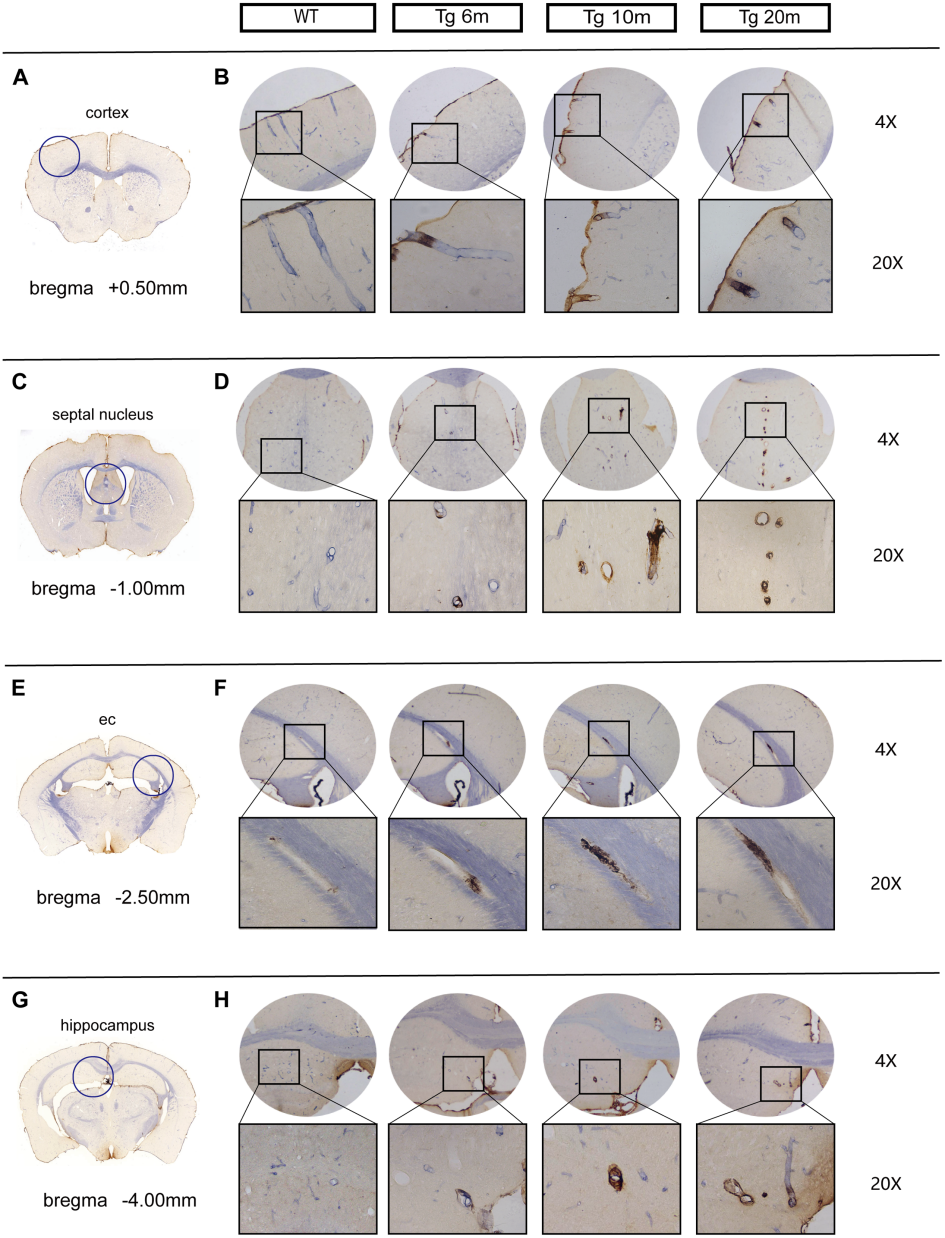
Deposition of NOTCH3^ECD^ in the brains of R75Q mouse: double-standard immunohistochemical staining exhibits tan (NOTCH3^ECD^) and blue-purple (SMC). **(A)** The level of coronal section of the brain is bregma +0.50 mm, and inside the circle is the cortex. **(B)** NOTCH3^ECD^ deposition in the cortex of different groups at the same level as **A**. **(C)** The level of coronal section of the brain is bregma −1.00 mm, and inside the circle is the septal nucleus (belong to subcortical nuclei). **(D)** NOTCH3^ECD^ deposition in the septal nucleus of different groups at the same level as **C**. **(E)** The level of coronal section of the brain is bregma −2.50 mm, and inside the circle is the external capsule (belong to white matter). **(F)** NOTCH3^ECD^ deposition in the external capsule of different groups at the same level as **E**. **(G)** The level of coronal section of the brain is bregma −4.00 mm, and inside the circle is the hippocampus. **(H)** NOTCH3^ECD^ deposition in the hippocampus of different groups at the same level as **G**.

**Figure 4:**
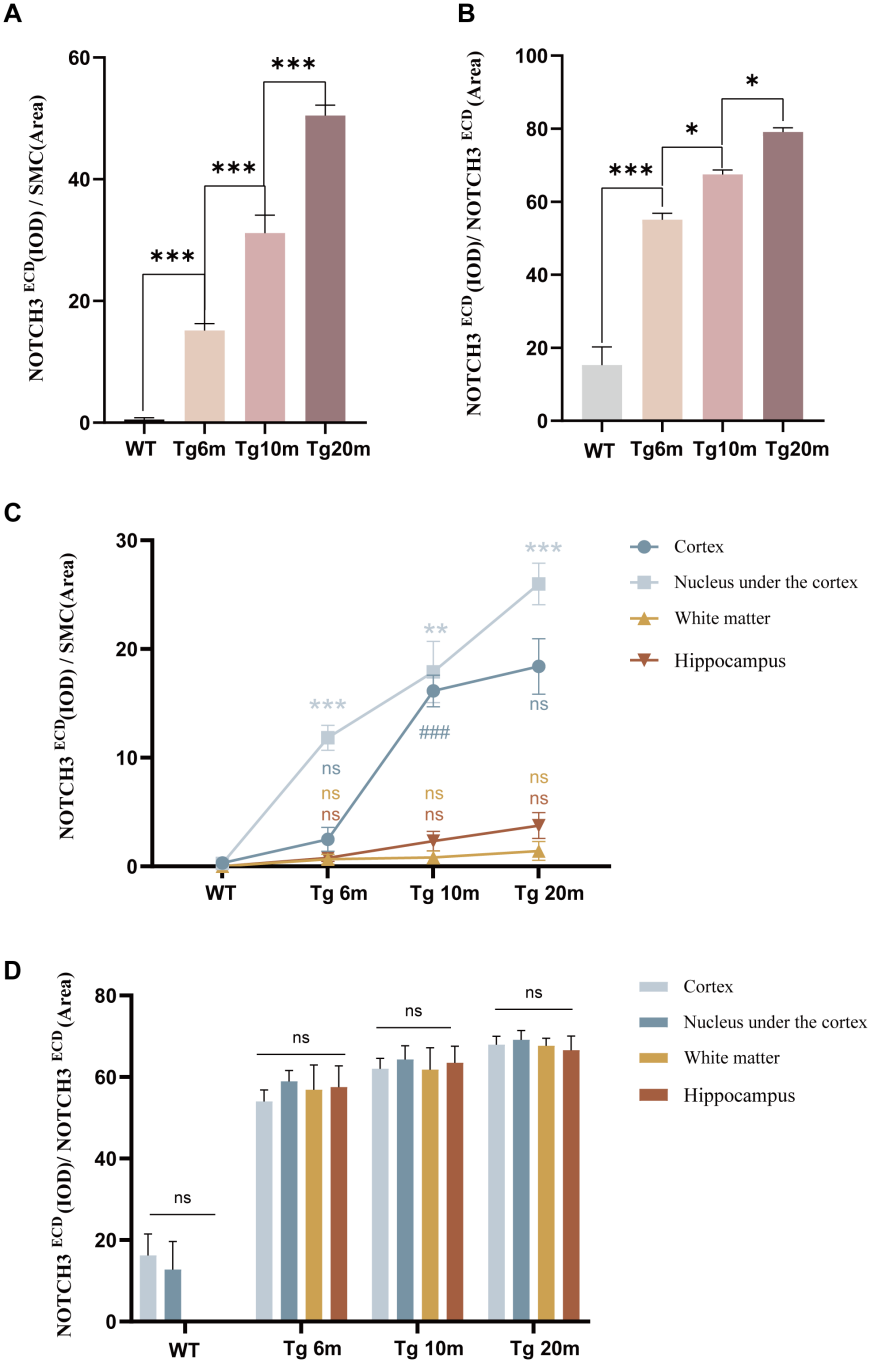
Quantitative analysis of NOTCH3^ECD^ deposition: **(A)** semi-quantitative analysis of the whole brain NOTCH3^ECD^ deposition rate, deposition rate = NOTCH3^ECD^ (IOD)/SMC (area). **(B)** Semi-quantitative analysis of the whole brain NOTCH3^ECD^ deposition degree, deposition degree = NOTCH3^ECD^ (IOD)/NOTCH3^ECD^ (area). **(C)** Semi-quantitative analysis of the different brain regions NOTCH3^ECD^ deposition rate. **(D)** Semi-quantitative analysis of the different brain regions NOTCH3^ECD^ deposition degree. Data are presented as means ± SD. ∗*p* < 0.05, ns *p* > 0.05, *n* = 4 per group. One—way ANOVA or Kruskal–Wallis *H* test, and Least-Significant Difference (*LSD*) was used for pairwise comparisons.

Double-labeling immunohistochemical staining of NOTCH3^ECD^ and smooth muscle cells in the brain revealed that NOTCH3^ECD^ accumulation was detected only in vascular smooth muscle in the Tg group (Figure 3), which is consistent with the deposition of NOTCH3^ECD^ in clinical CADASIL patients. To further observe the deposition characteristics of different brain regions, we selected four layers of the brain (bregma +0.50 mm, −1.00 mm, −2.50 mm, −4.00 mm), which included the cortex, subcortical nuclei, white matter, and hippocampus, and found that NOTCH3^ECD^ aggregates could be detected in different brain regions at four levels. This suggests that NOTCH3^ECD^ deposits accumulate in multiple brain regions throughout the brain (Figure 3). The deposition rate of NOTCH3^ECD^ aggregates in the cortex and subcortical nuclei were significantly higher than those in the white matter and hippocampus (*p* < 0.05) (Figure 4C). However, there was no significant difference in the degree of deposition of NOTCH3^ECD^ aggregates in the different brain regions (Figure 4D).

We further explored the effect of age on NOTCH3^ECD^ aggregation and found that the rate of NOTCH3^ECD^ deposition in the brain increased with age (*p* < 0.05) (Figure 4A), and the degree of NOTCH3^ECD^ deposition increased with age (*p* < 0.05) (Figure 4B). Therefore, we believe that age is a non-negligible factor affecting the accumulation of NOTCH3^ECD^ and is positively correlated with it.

#### 3.3.2 Characteristics of GOM deposition in the brain of R75Q mouse

**Figure 5:**
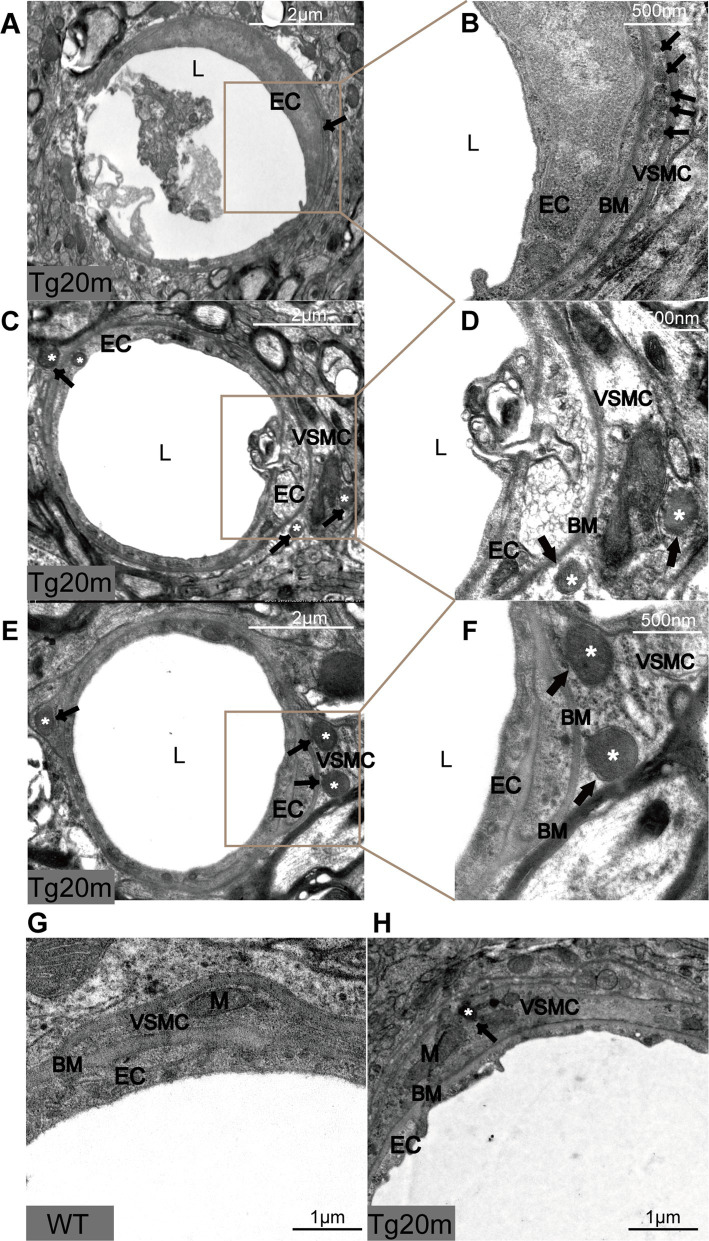
Pathological changes in R75Q mouse under electron microscope. L = lumen of blood vessels, EC = endothelial cell, BM = basement membrane, VSMC = vascular smooth muscle cell, M = mitochondrion, black arrows and white * mark GOM.

NOTCH3^ECD^ is the main component of GOM particles (Ishiko et al., 2006). In this mouse model, NOTCH3^ECD^ accumulated the most in the cortex and subcortical nuclei; therefore, we took samples from this area for electron microscopy and found grit-like dense osmiophilic granule (GOM) deposits on arterioles located near or at the membrane folds of VMSCs. Such granular deposits can also be observed at the basement membrane of some degenerated parietal cells, exhibiting different shapes and sizes as well as osmiophilic densities. Some small and round osmiophilic granules were located far from the cells, and the basement membrane was homogeneous and dense (Figures 5A,B). In some irregularly shaped GOMs, osmiophilic granules near the cells or basement membrane were dense (Figures 5C,D), while those far from the cells or basement membrane were loose and difficult to distinguish from the collagen material (Figures 5E,F).

In addition, we also observed the morphology of cerebral arteriolar smooth muscle cells (SMC) under electron microscopy. The cell membrane of VSMCs in the Tg group was shrunken, especially near the GOM. The cytoplasm of SMC had pigment accumulation, vacuole formation and mitochondrial volume increase. These changes were not observed in WT mice (Figures 5G,H). Thus, VSMC degeneration was more pronounced in the Tg group.

#### 3.3.3 Effect of *NOTCH3 R75Q* mutation on cell death

**Figure 6:**
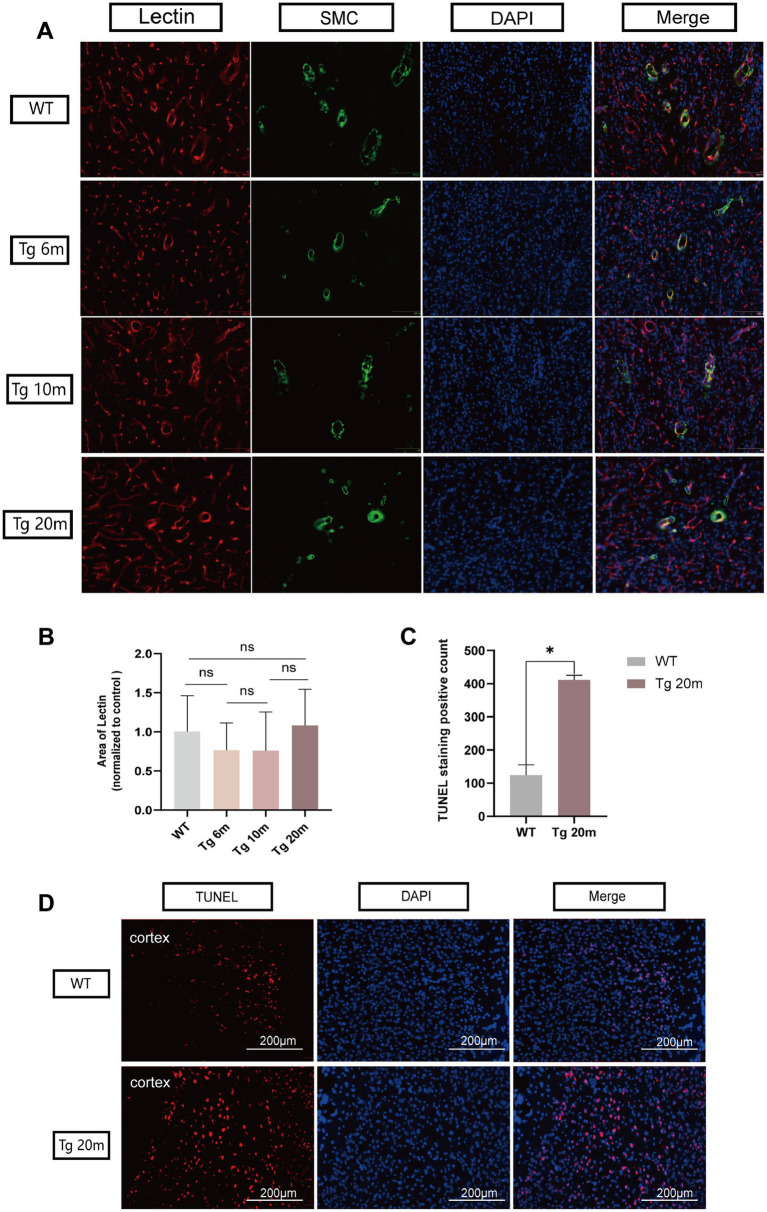
Results of lectin and TUNEL immunofluorescence staining of R75Q mice brain. **(A)** Immunofluorescence staining exhibits endothelial cells (red), SMC (green) and nuclei (blue) in mice brain. **(B)** Semi-quantitative statistical analysis, endothelial cell coverage in different experimental groups. **(C)** The positive cells in both groups were counted and statistically analyzed, and the data were finally normalized to the control group. **(D)** Immunofluorescence staining exhibits dead cells (red), and nuclei (blue) in mice brain. Data are presented as means ± SD. ∗*p* < 0.05, ns *p* > 0.05, *n* = 4 per group. One—way ANOVA or Kruskal–Wallis *H* test, and Least-Significant Difference (*LSD*) was used for pairwise comparisons.

The cellular and mouse model shows that *NOTCH3 R75Q* can result in aggregation of NOTHC3^ECD^ and GOM. To further demonstrate that this mutation can lead to apoptosis, TUNEL staining was performed on the brain tissue of R75Q and WT mice (Figure 6D). Dead cells appear red under fluorescent staining. We mainly found that apoptosis occurs in the cortex. This may be related to the fact that NOTCH3^ECD^ is mainly deposited in the cortex and subcortex. After counting the positive cells in mouse cerebral cortex and statistical analysis, the results showed that the degree of apoptosis in brain tissue of R75Q mice was higher than that of WT mice (*p* < 0.05) (Figure 6C). It can be demonstrated that the *NOTCH3 R75Q* mutation affects and promotes cell death.

#### 3.3.4 Characterization of cerebral small blood vessels in R75Q mouse

CADASIL is a hereditary disease involving cerebral small vessels (Yamamoto et al., 2021). Cerebral small vessels arise from the leptomeningeal vascular network formed by branches of cortical arteries in the subarachnoid space of the brain surface. There are penetrating arteries from the large vessels in the basal part of the brain. The two groups of penetrating arterioles pass through the cerebral cortex and deep gray matter nuclei, respectively, and then meet in the deep subcortical white matter area (Smirnov et al., 2021).

**Figure 7:**
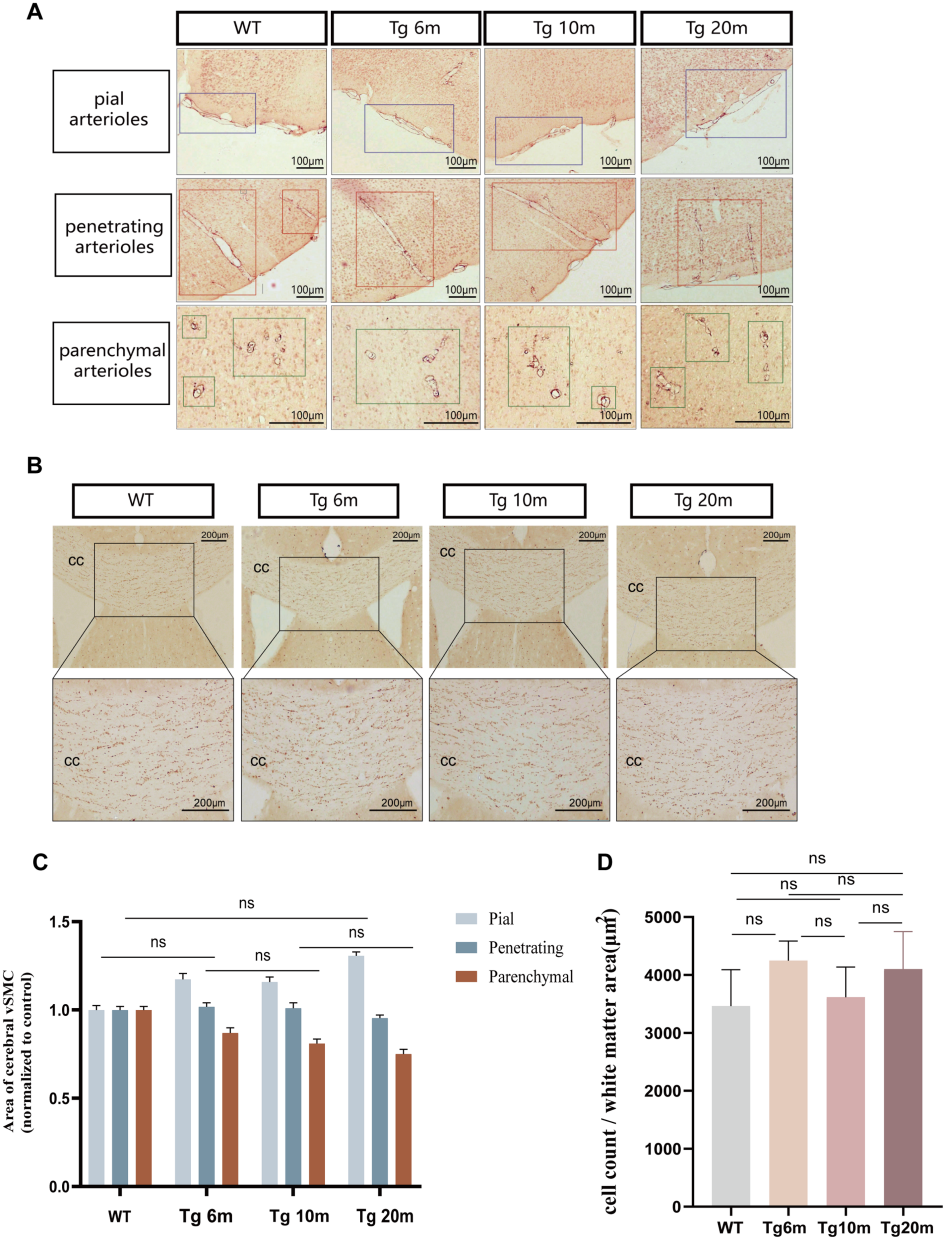
Phenotypic analysis of R75Q mouse (smooth muscle cells and white matter). **(A)** Immunohistochemical staining exhibits tan (SMC). The purple boxes are the corresponding groups of pial arteries. The red boxes are the corresponding groups of perforating arterioles. The green boxes are the corresponding groups of parenchymal arterioles. **(B)** Immunohistochemical staining exhibits tan (oligodendrocytes). **(C)** Semi-quantitative analysis of **A**. **(D)** Semi-quantitative analysis of **B**. Data are presented as means ± SD. ns *p* > 0.05, *n* = 4 per group. One—way ANOVA or Kruskal–Wallis *H* test, and Least-Significant Difference (*LSD*) was used for pairwise comparisons.

Immunohistochemical staining of brain VSMCs allowed us to observe the morphology and type of blood vessels. We mainly observed cerebral arterioles, including leptomeningeal arteries covering the surface of the cerebral cortex, perforating arterioles passing through the cortex, and arterioles passing through the brain parenchyma (Figure 7A). By quantitative analysis of SMC coverage area in different groups and different blood vessels, we found that the number of smooth muscle cells in different vascular morphology was not significantly different between the experimental group and the control group (*p* > 0.05) (Figure 7C) so there was no significant loss of smooth muscle cells in the cerebral arterioles of Tg mice.

To further characterize the vessels, we supplemented immunofluorescence staining of endothelial cells and smooth muscle cells of the vessels. *Lycopersicon Esculentum* (Tomato) Lectin (Thermo, United States) was used to label vascular endothelial cells and transgelin/SM22 Polyclonal antibody (Proteintech, United States) was used to label smooth muscle cells. The co-localization of endothelial cells and smooth muscle cells makes blood vessel morphology more characteristic (Figure 6A). It is also frustrating that after semi-quantitative statistical analysis of endothelial cells, no significant differences was observed in the coverage of endothelial cells between different groups (*p* > 0.05) (Figure 6B).

#### 3.3.5 R75Q mice had no significant damage to white matter

CADASIL is characterized by diffuse white matter lesions, especially deep white matter, and white matter lesions are mainly characterized by axonal demyelination resulting in loose white matter structure (Di Donato et al., 2017). The anatomy system of white matter in mice has not been well studied (Smirnov M, et al., 2021). Therefore, we focused on the corpus callosum, the deep white matter located between the subcortical and gray matter nuclei. Immunohistochemical staining marked oligodendrocytes, which were mainly involved in the constitutive of white matter, especially the myelination of axons and providing nutritional support and inflammation regulation for axons (Kuhn et al., 2019).

To observe the phenotype of R75Q mice, we used immunohistochemical labeling of oligodendrocytes to reflect the white matter of the brain. To observe the phenotype of R75Q mice, we used immunohistochemical labeling of oligodendrocytes to reflect the white matter of the brain (Figure 7B). There was no significant difference in oligodendrocyte density between control and experimental groups (*p* > 0.05) (Figure 7D), so we concluded that there was no significant damage to white matter in R75Q mice.

#### 3.3.6 Characteristics of NOTCH3^ECD^ aggregates in R75Q mouse skin

**Figure 8:**
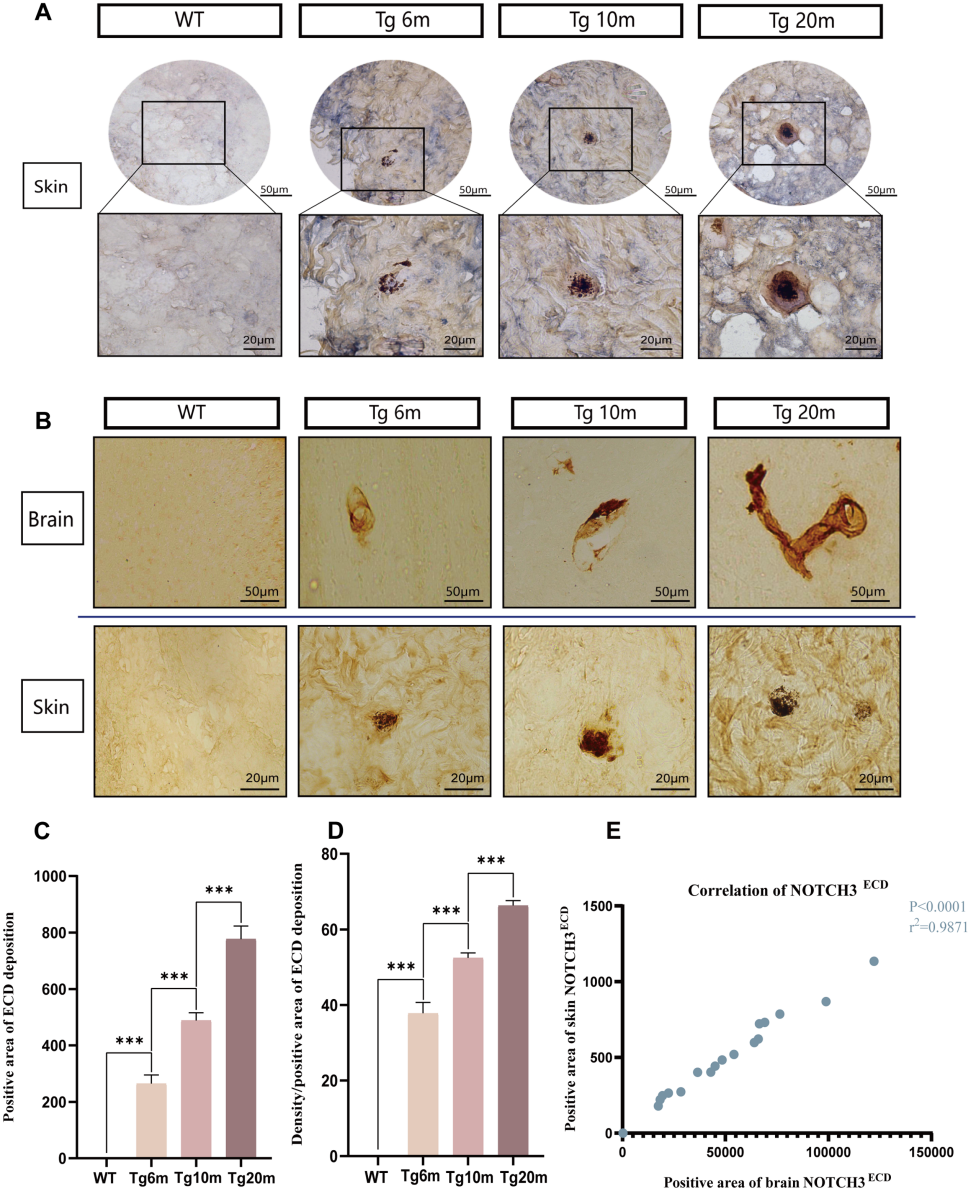
Analysis of NOTCH3^ECD^ aggregates in R75Q mouse skin. **(A)** Double-standard immunohistochemical staining exhibits tan (NOTCH3^ECD^) and blue-purple (SMC) in the skin. **(B)** Immunohistochemical staining exhibits tan (NOTCH3^ECD^) in the brain and skin. **(C)** Semi-quantitative analysis of the skin NOTCH3^ECD^ deposition rate, deposition rate = NOTCH3^ECD^ (area). **(D)** Semi-quantitative analysis of the skin NOTCH3^ECD^ deposition degree, deposition degree = NOTCH3^ECD^ (density)/NOTCH3^ECD^ (area). **(E)** Correlation analysis between skin and brain. Data are presented as means ± SD. ∗*p* < 0.05, *r*^2^ = 0.9871, *n* = 4 per group. One—way ANOVA or Kruskal–Wallis *H* test, and Least-Significant Difference (*LSD*) was used for pairwise comparisons. Pearson’s correlation coefficient was used to analyze the correlations between variables.

NOTCH3^ECD^, as a pathological marker of CADASIL, not only deposits in central tissues, but also can involve peripheral tissues. Double immunohistochemical staining of NOTCH3^ECD^ and SMC in mouse skin was performed. We found that NOTCH3^ECD^ in mice is not only expressed in cerebral vessels, but also in skin vessels (Figure 8A). Semi-quantitative statistical analysis of the deposition rate and degree of NOTCH3^ECD^ in skin tissue showed that the deposition rate and degree of NOTCH3^ECD^ progressed with age (*p* < 0.05) (Figures 8C,D), which was consistent with the deposition characteristics of NOTCH3^ECD^ in the brain (*p* < 0.001) (Figures 8B,E).

## 4 Discussion

In our study, the *NOTCH3 R75Q* mutant model was constructed for the first time both *in vitro* and *in vivo*. The *NOTCH3 R75Q* knock-in mouse model was the first cysteine-sparing mutant animal model, and based on *in vitro* cell experiments and *in vivo* transgenic mouse verification, we demonstrated that this site is pathogenic, which corresponds to previous clinical cases. In addition, the pathological molecular characteristics of cysteine-sparing mutations were explored, and the toxic effect and deposition characteristics of NOTCH3^ECD^ in *NOTCH3 R75Q* mutations were revealed.

To research the disease, the identification of feasible biomarkers is indispensable. White matter lesions in neuroimaging, although correlated with disease severity (Dichgans et al., 1999), do not apply to animal model studies. As the main pathogenic substance associated with CADASIL, NOTCH3 aggregation in blood vessels exists in all stages of disease progression (Rutten et al., 2015) and can even appear ten years earlier than clinical symptoms (Joutel et al., 2001; Lesnik Oberstein et al., 2003). The “NOTCH3 score” is used to quantitatively measure NOTCH3 in CADASIL model mice as a preclinical biomarker of CADASIL (Rutten et al., 2015). Therefore, NOTCH3 can also be used as a biomarker to assess pathogenicity in a cysteine-free mutation model.

We constructed *in vitro* NOTCH3^ECD^ R75Q mutant cell lines with lentivirus-stable strains. We also revealed that NOTCH3^ECD^ R75Q is resistant to protein degradation, which makes it easier to form abnormal aggregates of NOTCH3, which are cytotoxic. Therefore, in the cellular model, this mutation altered cellular function, further confirming the pathogenicity of the cysteine-sparing mutation, which is consistent with the *in vitro* cell studies by other researchers (Huang et al., 2020; Liu et al., 2022). Therefore, the abnormal aggregation of NOTCH3 and increased resistance to its degradation are common mechanisms in cysteine-sparing mutant cells.

Notably, current animal models of CADASIL are cysteine-mutant mice. For the first time, we constructed transgenic mice with cysteine-sparing mutations, which lays the foundation for future studies of this type, and our results show that the knock-in mouse model of R75Q mutation can simulate the pathological features of abnormal protein deposition in CADASIL. In our R75Q mice, the first accumulation of NOTCH3 was observed at 6 months of age, whereas GOM and VSMC degeneration occurred only in the cerebral arterioles at 20 months of age. This is similar to the relatively common CADASIL mutation model in R170C transgenic mice (Wallays et al., 2011). This model, along with R169C transgenic mice, is the only one to develop neuropathological changes, such as white matter degeneration and spontaneous infarction (Wallays et al., 2011; Joutel et al., 2010). Our results also showed a progressive increase in NOTCH3^ECD^ deposition with age, similar to the R182C transgenic mouse model, which is considered suitable for therapeutic strategies and preclinical testing to delay or reverse NOTCH3 accumulation (Rutten et al., 2015).

GOM is the gold standard for pathological diagnosis of CADASIL (Chabriat et al., 1998), therefore, it is also necessary to study the characteristics of GOM in a mouse model. Our results show a stochastic distribution of GOM, not only in the basement membrane, but also in the extracellular space adjacent to the plasma membrane of VSMCs, and at the folds of the plasma membrane of VSMCs. Because the R75Q mouse exhibited both NOTCH3^ECD^ and GOM deposition, TUNEL staining was used to further study the cell death. Although the results showed that the mutation promoted apoptosis, no significant loss was found in smooth muscle cells, endothelial cells, and oligodendrocytes. This is the same as in CADASIL transgenic mice expressing R90C, R182C, C428S, C455R, and R1031C cysteine mutations (Manini and Pantoni, 2021; Gravesteijn et al., 2020; Arboleda-Velasquez et al., 2011). Combined with the CADASIL transgenic mouse model, it is indeed not a good model to simulate and explore white matter damage. Several literature have provided models to study oligodendrocyte and white matter lesions. It can be divided into *in vivo* model and *in vitro* model. For example, immortalized oligodendroglial cell lines (CG-4, OLN-93, Oli-neu), derived from human iPSC, mouse models (Sox10—Venus mice, CNP—EGFP mice and PLP—EGFP mice) and zebrafish models [Tg (sox10:mRFP), Tg (olig2:EGFP), and Tg (mbp:EGFP)]. It is speculated that significant differences in the cerebral vessels of mice may reduce their susceptibility to hypoperfusion, so that phenotypes are less likely to emerge (Manini and Pantoni, 2021). In addition, Oligodendrocytes, which constitute axons, and smooth muscle cells, which constitute blood vessels, have more functions. They can differentiate into different phenotypes in response to changes in internal and external environment. Therefore, the R75Q mutation does not necessarily cause their death. Labeling these cells with a single protein, then, would not fully reveal their pathological changes.

In addition, NOTCH3^ECD^ deposition in the skin was age-dependent, consistent with the brain trend, which illustrates that deposition in the periphery reflects that in the brain. In CADASIL, the major skin related pathological changes were NOTCH3 protein positive and GOM particle positive. Both of them serve as pathological markers of CADASIL, making skin biopsy one of the diagnostic criteria for CADASIL. CADASIL related skin disease is extremely rare. Only one case was reported in the literature 20 years ago that a case of CADASIL with generalized haemorrhagic macules and patches. In addition to the typical neurological symptoms and histopathological results confirmed the diagnosis. Immunofluorescence also showed an increased number of blood vessels and a marked thickening of the vessel wall due to deposition of fibrin, complement, and immunoglobulin (Ratzinger et al., 2005). In addition, some researchers have observed mild neurovascular disorders in the skin by assessing the somatic and autonomic nerves of the skin (Nolano et al., 2016). Other studies have shown that poor vascular reactivity is found in patients with induced CADASIL when they are given skin vasoconstriction and relaxation (Gobron et al., 2007). Although these peripheral pathological changes are not obvious, they all reflect the influence of the disease on microvessels to a certain extent.

There are still many directions worth investigating about CADASIL. Such as, in the process of regulating cell classification and apoptosis, inflammation factors and inflammatory cells may also be involved (Colonna and Butovsky, 2017). Microglia and astrocytes play a role in neuroinflammation in vascular dementia and neurodegenerative diseases (Kwon and Koh, 2020). At present, there are not many inflammatory studies on CADASIL. Perhaps, from the perspective of inflammation, the process of cell classification and apoptosis can be effectively studied. And the construction of transgenic mouse models can not only observe pathological changes, but also observe and study through behavioral cognition. In the current CADASIL mouse model, only the R170C mutant mice have significant behavioral changes (Wallays et al., 2011). R170C Mice were monitored during aging for spontaneous motor abnormalities (reduced spontaneous mobility, tremor, ataxia as observed by staggering gait, and limb paresis as observed by dragging of a limb when stimulated to walk on a tabletop). However, there are no behavioral studies on mood, depression, cognitive impairment, etc. This would be a promising research direction.

In conclusion, our study revealed the toxic role of NOTCH3^ECD^ in *NOTCH3 R75Q* mutation both *in vitro* and *in vivo* models and demonstrated that NOTCH3^ECD^ can also serve as a biomarker for the pathogenicity of cysteine-sparing mutations. In addition, cysteine-sparing mutant mouse model has been constructed for the first time to simulate the pathological features of abnormal protein aggregation in CADASIL. This study lays the foundation for further research on the pathogenesis and therapeutic intervention strategies.

## Data Availability

The original contributions presented in the study are included in the article/Supplementary material, further inquiries can be directed to the corresponding authors.
