# Supplementary material for: Analysis of the pathogenicity and pathological characteristics of NOTCH3 gene-sparing cysteine mutations in vitro and in vivo models
Source: Front Mol Neurosci. 2024 Dec 20;17:1391040. doi: 10.3389/fnmol.2024.1391040 (PMC11695339; doi:10.3389/fnmol.2024.1391040)
Supplement: Supplementary file 1 [file Table_1.docx]

Supplementary Material

# Supplementary Data

Gene knock-in mice appraisal designed primers：

PCR Primers WT (Annealing Temperature 60.0 ºC):

F: 5’-CTCTACTGGAGGAGGACAAACTG-3’:

R: 5’-GTCTTCCACCTTTCTTCAGTTAGC-3’ ;Wildtype allele: 479 bp

PCR Primers *R75Q*(Annealing Temperature 60.0 ºC):

F: 5’-TGCTAACCATGTTCATGCCTTCTT-3’

R: 5’-CACTCATCCACGTCGCTTCG-3’ ;R75Q *allele*: 375bp。

Immunofluorescence staining and immunohistochemical staining of the involved antibodies：

①Anti-NOTCH3/N3ECD,clone 1E4;(Tikka et al., 2012)

②Anti-NOTCH3 antibody [EPR25183-101](AB300527);

③transgelin/SM22 Polyclonal antibody 10493-1-AP|proteintech;

④[Anti-Olig2 antibody [EPR2673] (ab109186)](https://www.abcam.cn/products/primary-antibodies/olig2-antibody-epr2673-ab109186.html)

⑤Goat Anti-Mouse IgG H&L(Alexa Fluor^Ⓡ^594)(ab150116); (Oliveira et al., 2023)

⑥Goat Anti-Mouse IgG H&L(Alkaline Phosphatase) preadsorbed(ab7069);

⑦Goat Anti-Rabbit IgG H&L(HRP)(ab6721);

⑧Goat Anti-Mouse IgG H&L(HRP)(ab6789);

# References

# Tikka, S., Ng, Y. P., Di Maio, G., Mykkänen, K., Siitonen, M., Lepikhova, T., Pöyhönen, M., Viitanen, M., Virtanen, I., Kalimo, H., & Baumann, M. (2012). CADASIL mutations and shRNA silencing of NOTCH3 affect actin organization in cultured vascular smooth muscle cells. *Journal of cerebral blood flow and metabolism : official journal of the International Society of Cerebral Blood Flow and Metabolism*, *32*(12), 2171–2180. https://doi.org/10.1038/jcbfm.2012.123

Oliveira, D. V., Coupland, K. G., Shao, W., Jin, S., Del Gaudio, F., Wang, S., Fox, R., Rutten, J. W., Sandin, J., Zetterberg, H., Lundkvist, J., Lesnik Oberstein, S. A., Lendahl, U., & Karlström, H. (2023). Active immunotherapy reduces NOTCH3 deposition in brain capillaries in a CADASIL mouse model. *EMBO molecular medicine*, *15*(2), e16556. https://doi.org/10.15252/emmm.202216556
